# Wake EEG oscillation dynamics reflect both sleep need and brain maturation across childhood and adolescence

**DOI:** 10.1101/2024.02.24.581878

**Published:** 2024-02-28

**Authors:** Sophia Snipes, Elena Krugliakova, Valeria Jaramillo, Carina Volk, Melanie Furrer, Mirjam Studler, Monique LeBourgeois, Salome Kurth, Oskar G. Jenni, Reto Huber

**Affiliations:** 1 Child Development Center, University Children’s Hospital Zurich, University of Zurich, Zurich, Switzerland; 2 Donders Institute, Radboud University Medical Center, Nijmegen, the Netherlands; 3 School of Psychology, University of Surrey, Guildford, UK; 4 Surrey Sleep Research Centre, Faculty of Health and Medical Sciences, University of Surrey, Guildford, United Kingdom; 5 UK Dementia Research Institute Care Research and Technology Centre, Imperial College London and the University of Surrey, Guildford, UK; 6 Department of Social Neuroscience and Social Psychology, Institute of Psychology, University of Bern, Bern, Switzerland; 7 University of Colorado at Boulder, Department of Integrative Physiology, Boulder, Colorado, USA; 8 The Warren Alpert Medical School of Brown University, Department of Psychiatry and Human Behavior, Providence, Rhode Island, USA; 9 In memoriam; 10 Department of Psychology, University of Fribourg, Fribourg, Switzerland; 11 Department of Pulmonology, University Hospital Zurich, Zurich, Switzerland; 12 Department of Child and Adolescent Psychiatry and Psychotherapy, Psychiatric Hospital, University of Zurich, Switzerland

## Abstract

An objective measure of brain maturation is highly insightful for monitoring both typical and atypical development. Slow wave activity, recorded in the sleep electroencephalogram (EEG), reliably indexes changes in brain plasticity with age, as well as deficits related to developmental disorders such as attention-deficit hyperactivity disorder (ADHD). Unfortunately, measuring sleep EEG is resource-intensive and burdensome for participants. We therefore aimed to determine whether wake EEG could likewise index developmental changes in brain plasticity. We analyzed high-density wake EEG collected from 163 participants 3–25 years old, before and after a night of sleep. We compared two measures of oscillatory EEG activity, amplitudes and density, as well as two measures of aperiodic activity, intercepts and slopes. Furthermore, we compared these measures in patients with ADHD (8–17 y.o., N=58) to neurotypical controls. We found that wake oscillation amplitudes behaved the same as sleep slow wave activity: amplitudes decreased with age, decreased after sleep, and this overnight decrease decreased with age. Oscillation densities were also substantially age-dependent, decreasing overnight in children and increasing overnight in adolescents and adults. While both aperiodic intercepts and slopes decreased linearly with age, intercepts decreased overnight, and slopes increased overnight. Overall, our results indicate that wake oscillation amplitudes track both development and sleep need, and overnight changes in oscillation density reflect some yet-unknown shift in neural activity around puberty. No wake measure showed significant effects of ADHD, thus indicating that wake EEG measures, while easier to record, are not as sensitive as those during sleep.

## INTRODUCTION

The EEG is one of few tools available to study the human developing brain already from birth (Korotchikova et al., 2009). It is non-invasive, relatively cheap, and provides a real-time readout of neuronal activity. It is an incredibly rich signal, with the potential as a prognostic and diagnostic tool for both typical development and disease. Sleep EEG, and in particular slow wave activity (SWA, 0.5–4 Hz) during NREM sleep, has proven especially sensitive to brain maturation (Campbell & Feinberg, 2009) and developmental disorders such as ADHD (Furrer et al., 2019). This is because SWA reflects the overall synchronicity of the brain, which decreases with age following decreasing synaptic density across adolescence (Campbell & Feinberg, 2009; Huttenlocher, 1979; Jenni & Carskadon, 2004), and may be lower in ADHD due to reduced cortical thickness (Shaw et al., 2006). Furthermore, SWA is greater in occipital regions in younger children, and greater in frontal regions in adolescents and adults (Kurth et al., 2010), possibly reflecting the slower maturation of higher order association areas (Shaw et al., 2008).

In addition to these developmental phenomena, SWA reflects the buildup and dissipation of sleep need, increasing following wake and decreasing during sleep (Borbély, 1982). These hourly changes in SWA are hypothesized to reflect synaptic *plasticity*: synaptic strength increases with wake and daytime learning and decreases during sleep (Tononi & Cirelli, 2003, 2014). Generally, plasticity decreases with brain maturation across childhood and adolescence and this is reflected in decreases in the daytime and overnight *changes* in SWA with age (Jaramillo et al., 2020). Thus, both absolute SWA and changes in SWA are markers of brain development, reflecting synaptic density and synaptic plasticity respectively.

Sleep data is highly informative but can be burdensome to collect, involving either a sleep laboratory or at-home measurements, somewhat uncomfortable sleeping conditions which can affect sleep quality, and several hours of recordings to process. Wake EEG is much more accessible, typically measured in the span of minutes to a couple hours. Traditionally, EEG during both sleep and wake is quantified as spectral power, which summarizes a time signal by indicating how much of any given frequency is present in that signal (Cohen, 2014). However, greater insights into neuronal activity can be derived from more specific analyses (Donoghue et al., 2020).

**Figure 1:**
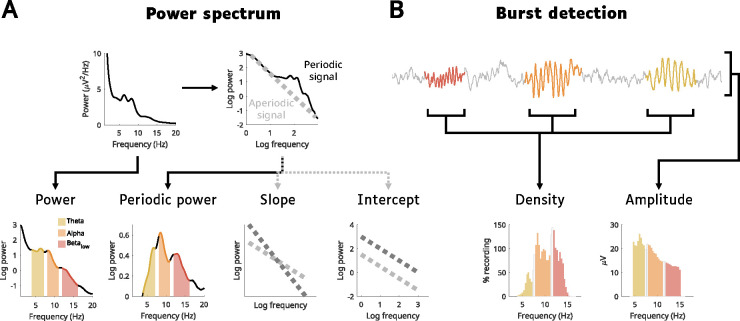
EEG outcome measures. **A**: Measures based on spectral power. Given the nature of power, it is traditionally analyzed log-transformed to have more normally distributed values. These are then aggregated into bands. Here, we focus on classical wake EEG bands: theta (4–7 Hz, yellow), alpha (8–11 Hz, orange) and low beta (12–16 Hz, red), with gaps between bands to avoid overlapping information. The example comes from a 15-year-old male participant, used for the entire figure. The EEG signal is composed of aperiodic “background activity” (gray parts in B) and oscillatory activity (colored parts in B). When plotting the power spectrum on a log-log scale, a line can be fitted to the aperiodic component of the signal, which can be subtracted from the whole spectrum, leaving behind only periodic power. The aperiodic line can then be quantified by its intercept (where it intersects 0 on the log-log scale, i.e. the log power at 1 Hz), and its slope (how tilted it is). Thus, the power spectrum provides four main outcome measures: log-transformed power, periodic power, slope and intercept of the aperiodic signal. **B**: Cycle-by-cycle analysis is used to detect bursts of oscillations (see methods) by identifying sections of the EEG signal that show periodic activity (colored), relative to the aperiodic background activity (gray sections). Once bursts are detected, there are two main parameters to quantify them: density (how much of the signal in time contains an oscillation) and amplitude (average peak-to-peak voltage of each oscillation). Densities are expressed in percentage, and when pooling bursts detected in all channels, they can easily exceed 100%, as the same burst will appear in multiple channels. Amplitudes are in microvolts. The EEG trace was stitched together for illustrative purposes. N.B. beta periodic power is lower than alpha periodic power (in panel A), but their densities (in B) are roughly the same; this is because periodic power is also influenced by the lower amplitudes of low beta compared to alpha.

The EEG is made up of both *periodic* activity and *aperiodic* activity (Figure 1A). Periodic activity refers to oscillations, which appear as quasi-gaussian bumps in the power spectrum at their corresponding frequency. Instead, aperiodic activity is a form of background “noise,” producing the characteristic 1/f curve in the EEG power spectrum. Aperiodic activity is defined by its *intercept* (reflecting the overall aperiodic power) and *slope* (the steepness of the curve), both of which can change across development, conscious states, and pathology (Bódizs et al., 2021; Cellier et al., 2021; Colombo et al., 2019, 2023; Favaro et al., 2023; Hill et al., 2022; Horváth et al., 2022; Ostlund et al., 2021; Robertson et al., 2019; Schneider et al., 2022; Tröndle et al., 2022). Changes in slope in particular are hypothesized to reflect alterations in excitatory/inhibitory balance of neuronal activity (Gao et al., 2017).

Aperiodic activity is quantified with spectral power. Periodic activity can likewise be quantified with power, by simply subtracting the aperiodic activity from overall power (giving periodic power, Figure 1A), however this misses two important independent changes that can happen to oscillations: they can change in *amplitude*, and they can change in *density* (Figure 1B). The amplitudes of oscillations reflect the synchronicity of the oscillating neuronal population, and that synchronicity is determined both by the number of neurons in phase with each other and the strength of their synaptic connections. Whether an oscillation occurs at all (i.e., density) will instead depend on the activity of “pacemaker” interneurons which entrain a population of neurons to the same rhythm (Le Bon-Jego & Yuste, 2007; Perkel et al., 1964), and this activity will be in service of some underlying function that will come and go as needed. In short, amplitude reflects synchronicity and density reflects activity.

Since oscillation amplitudes reflect synchronicity, this means they should reflect the same information as SWA measured during sleep. Supporting this, in an extended wake study in young adults, we found that wake oscillation amplitudes increased along a saturating exponential with time awake, and decreased following sleep (Snipes et al., 2023), thus following the same trajectories as SWA (Borbély, 1982; Dijk et al., 1987). Instead, theta oscillation densities (4–8 Hz) increased linearly with wake, and alpha oscillation densities (8–12 Hz) decreased, supporting the specificity of the effect to amplitudes, and masking the relationship to sleep need when only evaluating spectral power.

Given these results, we hypothesized that wake oscillation amplitudes should behave like sleep slow waves also across development: absolute amplitudes should decrease with age, and overnight changes in amplitude should decrease with age. Likewise, changes in amplitudes should also manifest an anterior-posterior gradient with age: larger amplitudes in occipital regions in children and larger in frontal regions in adults. Finally, given that children with ADHD have lower sleep SWA than age-matched controls, they should likewise have lower wake amplitudes. In short, if both wake amplitudes and sleep slow waves are supposed to reflect brain-wide synchronicity, then wake amplitudes should also reflect sleep need, brain development, and the pathophysiology of ADHD. We further hypothesized that all these effects would be specific to amplitudes, with other wake EEG parameters changing independently with sleep and age.

To answer these questions, we analyzed data collected from previous studies at the University Children’s Hospital of Zurich, with high-density wake EEG recordings measured the evening before and morning after a night of sleep. The final dataset included 105 neurotypical participants from the ages of 3.5 to 25, and 58 participants with ADHD (details in Table 1 and Table 2).

## RESULTS

### Wake oscillation amplitudes decrease with both sleep and age

**Figure 2:**
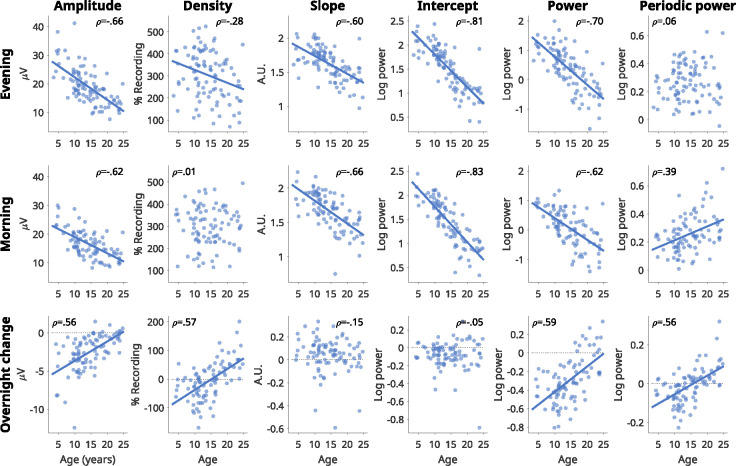
Wake EEG measures correlated with age. Only auditory oddball recordings are included, pooling both neurotypical and ADHD participants. Each dot represents the outcome value for a single participant. For participants with multiple sessions, values across sessions were first averaged. Pearson’s correlations were done for each figure, with rho values provided in the corner. If the p-value was less than .05, a correlation line was drawn (without correcting for multiple comparisons). Amplitudes and densities of oscillations were obtained from burst clusters, pooling all frequencies (4–16 Hz). Slope values are inverted, such that larger values indicate steeper slopes. Power spectra were calculated for each channel, then averaged across all channels, excluding the outermost ring of channels. Power and periodic power were then calculated by averaging values from 4 to 16 Hz.

To determine whether any wake EEG measure was related to development, sleep or ADHD, we conducted linear mixed effects models for each measure (amplitude, density, slope, intercept), pooling data across channels and frequencies (4–16 Hz; the range of most oscillatory activity). The same models were conducted for power and periodic power to evaluate the extent to which these summary measures followed similar trajectories. We had as fixed factors *Task* (oddball vs. go/no-go, alertness & fixation), *Sleep* (evening before sleep vs. morning after), *Age*, Sleep by Age interaction, *Group* (controls vs. ADHD), and *Sex* (female vs. male), and nested mixed factors *Session* and *Participant*. The full outputs of the models are provided in Suppl. Data 1. β estimates of continuous variables (e.g., age) indicate by how much the EEG “outcome” measure changes for each unit of the continuous variable (e.g., 1 year) when all other variables are 0. Similarly, the estimates of categorical variables (e.g. group) indicate how much the EEG measure changes from that category (e.g., ADHD) to the baseline category (e.g., controls), for all other factors set to 0. T-values allow a comparison of the magnitude of the effect of each factor. To visualize the main effects of age, Figure 2 provides the correlation between participants and each EEG measure of the oddball task (the most common task in the widest age range) in the evening and morning, as well as the overnight change. Rho values are provided in each plot as effect sizes.

As predicted, oscillation amplitudes significantly decreased with age, decreasing by 0.79 μV per year older (beta = −0.783, t = −11.33, p < .001, df = 1234). Amplitudes decreased significantly following sleep (beta = −3.977, t = −15.90, p < .001, df = 1234), with a significant positive interaction (beta = 0.143, t = 7.74, p < .001, df = 1234), such that amplitudes decreased less overnight with increasing age (Figure 2, bottom row). Amplitudes were lower in males than females (beta = −1.476, t = −2.38, p = .017, df = 1234), and were not significantly different in participants with ADHD (beta = −0.574, t = −0.89, p = .373, df = 1234). As can be seen in Figure 2, the relationship between age and amplitudes was quite robust, both as absolute values (ρ_eve_ = −.66, ρ_mor_ = −.63) and overnight changes (ρ = .56). Overall, amplitudes matched our predictions, except for ADHD: they were larger in young children, and the overnight change was larger in young children.

Oscillation density significantly decreased with age (beta = −3.540, t = −2.11, p = .035, df = 1234), and with sleep (beta = −77.506, t = −10.39, p < .001, df = 1234), with a significant positive interaction between age and sleep (beta = 4.878, t = 8.85, p < .001, df = 1234). Unlike amplitudes, the correlation between age and density was weak (ρ_eve_ = −.28, ρ_mor_ = .01). Instead, the correlation between age and overnight change in density was quite strong (ρ = .57), such that oscillation densities decreased overnight in children under 15 and increased in adults (Figure 2). There was no effect of ADHD (beta = −0.154, t = −0.01, p = .992, df = 1234) or sex (beta = −16.446, t = −1.10, p = .271, df = 1234). Overall, oscillation density behaved independently from amplitudes, especially in the direction of overnight changes in adolescents and adults.

Aperiodic slopes became significantly shallower with age (beta = −0.027, t = −9.23, p < .001, df = 1234) but significantly steeper overnight (beta = 0.099, t = 5.51, p < .001, df = 1234), with a trending negative interaction between sleep and age (beta = −0.003, t = −1.91, p = .056, df = 1234). The correlations between slopes and age were as robust as for oscillation amplitudes (ρ_eve_ = −.60, ρ_mor_ = −.66), but the correlation with overnight change was weak (ρ = −.15). There was no significant effect of ADHD (beta = 0.028, t = 1.07, p = .284, df = 1234) or sex (beta = 0.041, t = 1.63, p = .102, df = 1234). This means that, unlike for amplitudes, the overnight change goes in the opposite direction as the change with age.

Aperiodic intercepts also significantly decreased with age (beta = −0.067, t = −16.59, p < .001, df = 1234), but with no significant effect of sleep (beta = 0.029, t = 1.65, p = .100, df = 1234), and a significant negative interaction (beta = −0.004, t = −3.11, p = .002, df = 1234), such that intercepts decreased more overnight with age. The correlations between age and intercepts were the strongest of all outcome measures (ρ_eve_ = −.81, ρ_mor_ = −.83), however, the correlation between age and overnight change in intercept was negligible (ρ = −.05). Again, there was no effect of ADHD (beta = 0.021, t = 0.55, p = .579, df = 1234) or sex (beta = −0.003, t = −0.07, p = .944, df = 1234). Overall, average intercepts correlated with age in the same direction as amplitudes, changed overnight in the same direction, but the relationship between overnight change and age tended to be larger in adults than children.

In summary, of the four EEG measures, only amplitudes followed the same trajectories as SWA in sleep. The absolute values of all four measures had a negative correlation with age, and differed primarily in the overnight response and the relationship between age and overnight response. Oscillation densities in particular showed a strong effect of age on overnight changes, reversing direction between childhood and adolescence. No measure showed any relationship with ADHD, and only amplitudes were affected by sex. Therefore, in later analyses we did not include these factors, and pooled patients and controls for greater statistical power.

To determine the extent to which spectral power was influenced by any of the four main measures, and to quantify their interdependency, we compared each measure to the other and to average power and periodic power. We did this first by directly correlating all measures with each other, pooling all recordings (Suppl. Figure 1–1). Then, we ran linear mixed effects models to determine the t-values (as a proxy for effect size) of the fixed effect of one measure when predicting the other, controlling for the fixed effects of Task, Sleep, Age, Sleep interacting with Age, and the mixed effects Participant and Session (Suppl. Table 1–1). With both analyses, we found that power was most correlated with oscillation amplitudes (correlation ρ =.9; mixed model t = 44.4), whereas periodic power was most correlated with oscillation densities (ρ = .85; t = 50.4). Intercepts and slopes were most highly correlated with each other (ρ = .87; t = 63.9), and slopes were the least correlated with the other EEG measures.

### Each outcome measure showed unique regional effects, changing across age and sleep

**Figure 3:**
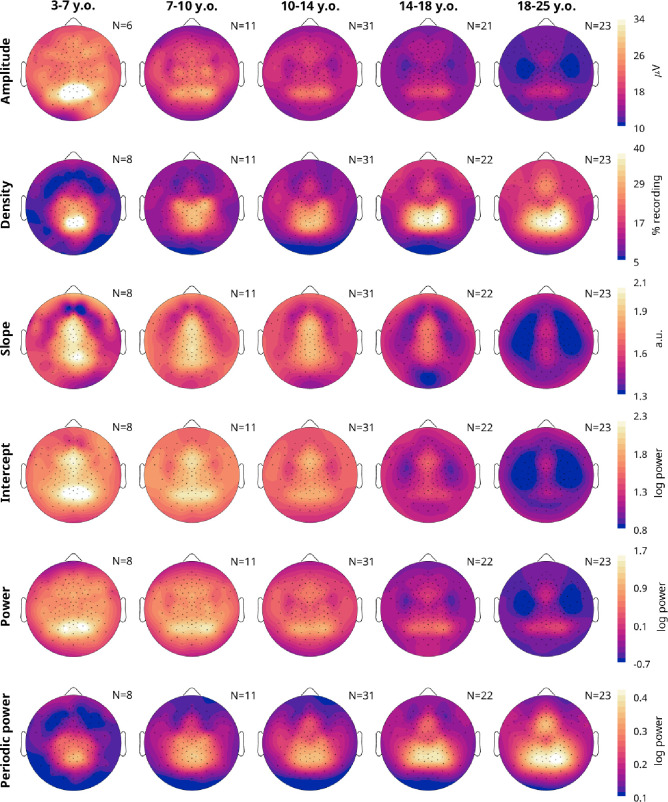
Topography averages of wake EEG measures. Each plot is a schematic of the EEG viewed from above, with the nose on top. Lighter colors indicate greater magnitude over a given location for that outcome measure (rows). Only neurotypical participants and oddball recordings were included, and participants were grouped into age bins (columns). Multiple oddball recordings from different sessions and times of day were first averaged for each participant. The number of participants included is indicated in the top right corner of each plot. Acronyms: y.o, years-old.

Figure 3 provides the average topographical maps of each measure for five age bins, averaging (or pooling for densities) all frequencies from 4–16 Hz, from the oddball task. Amplitudes, densities, slopes, and intercepts all showed unique topographies from each other. Across ages, for each measure there were primarily changes in magnitude more so than major regional differences. However, oscillation amplitudes in the youngest cohort began as a single midline occipital spot, which spreads bilaterally in the 7–10-year-olds. Prominent central bilateral peaks also appeared in the 7–10-year-olds. Oscillation densities similarly started as a single midline occipital spot, but these became more lateral-parietal in the 14–18-year-olds. Like amplitudes, two small bilateral central peaks emerge in the 7–10-year-olds, which merged with the primary occipital-parietal cluster in the 14–18-year-olds. Furthermore, a frontal peak gradually emerged with age. Slopes were steepest in midline channels, whereas intercepts showed both a frontal midline and occipital peak. As with the correlations between measures, power topographies most resemble amplitudes, and periodic power resembles densities.

**Figure 4:**
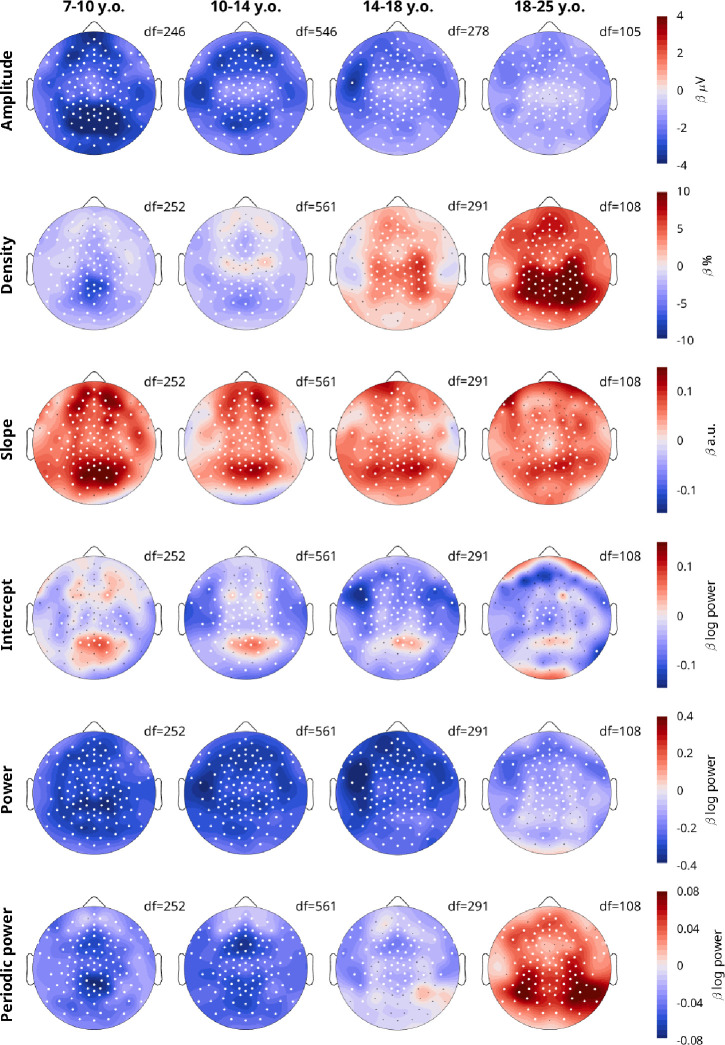
Topographies of overnight changes of EEG measures. A linear mixed effects model was run for each measure and each age group: Measure~Sleep+Task+(1|Participant)+(1|Participant:Session). Color reflects β estimates for the fixed effect *Sleep*, such that red indicates an overnight increase in that outcome measure. The factor *Task* was not included for the 18–25 y.o. group, as these participants only performed oddballs. White dots indicate channels for which the β estimate was statistically significant, corrected for multiple comparisons with FDR (false discovery rate). Black dots indicate remaining channels. Data includes both patients and neurotypical controls. Degrees of freedom (DF) are provided for each plot.

To determine the topography of overnight changes in EEG measures, we performed linear mixed effects models for each channel, dividing participants into 4 age bins (the youngest 3–7 were excluded as they were too few, with too few recordings). Fixed effects were Sleep and Task, mixed effects were Participant and Session. Figure 4 plots the β estimates for the effect of Sleep.

Amplitudes showed widespread overnight decreases across all age groups, however the decrease was largest in occipital channels for the youngest group, and slightly more fronto-temporal in young adults. Based on the relative β estimates, the decrease in all channels in children was larger than for adults.

The overnight density topographies resembled the average density topographies from Figure 3, in terms of regional effects. The youngest group showed the largest overnight *decrease* in the same midline-occipital spot where there were the most oscillations (Figure 3), and adults showed the largest *increase* in the same bilateral occipital-parietal areas where they had the largest densities.

The overnight increase in the steepness of slopes peaked in an occipital spot in all age groups, with additional bilateral frontal spots in <14-year-olds. These topographies do not correspond to the average topography of slopes from Figure 3. Intercepts revealed widespread decreases, with localized increases in the same occipital locations for which slopes increased the most. This suggests that aperiodic intercepts generally decrease, although the increase in slope steepness contrasts this effect.

Power and periodic power again showed similarities to amplitudes and densities respectively, however the increase in densities in the 14–18-year-olds was not visible in periodic power. Likewise, the overnight increase in periodic power for adults was more occipital and lateral than the increase in densities and the larger decrease in amplitudes in occipital regions was less evident in power than for amplitudes.

Overall, the topographical overnight changes extend the results from Figure 2: amplitudes decrease less with age, densities switch from decreasing to increasing before adolescence, slopes generally increase with sleep, and intercepts decrease. The posterior-anterior gradient of peak overnight decreases in amplitude further supports the similarity between wake amplitudes and sleep SWA. The regional differences between overnight decreases in density in children and increases in adults suggest that these oscillations not only originate from different areas but are functionally distinct.

### Overnight changes in density depend on the frequency of oscillations

**Figure 5:**
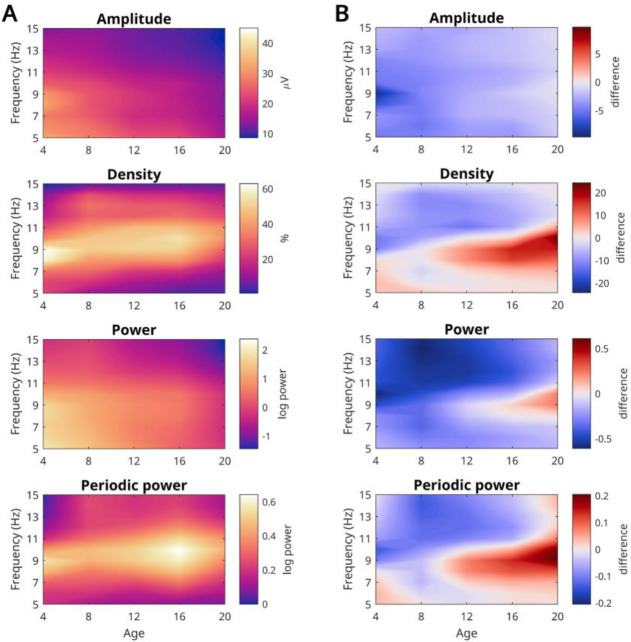
Average spectrograms of outcome measures. From the oddball task, pooling controls and ADHD participants. **A**: Average values, such that lighter colors indicate greater magnitude for a given frequency and age. For spectra extending to older ages and sleep stages, see Sun et al. (2023). **B**: Difference values between morning and evening recordings, such that red indicate a greater magnitude in the morning. The measurement unit of each figure is the same as that of A.

In the previous analyses, we had pooled all frequencies between 4 and 16 Hz. Here, we explored how each EEG measure changed for each frequency, this time averaging channels (or pooling, for densities). Average evening values for each age and frequency are plotted in Figure 5A, and the overnight differences are plotted in Figure 5B.

Amplitude and power showed gradual gradients, with highest amplitudes in the youngest participants and lowest frequencies, and lowest values in the oldest participants and highest frequencies. Density and periodic power instead had distinct peak values between 8 and 11 Hz, with the peak shifting upwards with age, a well-known property of alpha oscillations during development (Freschl et al., 2022; Smith, 1938; Tröndle et al., 2022).

Like average amplitudes, overnight changes in amplitude (and power) showed largely gradual decreases with age and frequency. Instead, like average densities, overnight densities (and periodic power) showed decreases in higher frequencies (>11 Hz, i.e. low beta) and increases in alpha (8–12 Hz). These increases only began between 8–10 years of age, they were strongest in adults, and the range shifted to higher frequencies with age.

**Figure 6:**
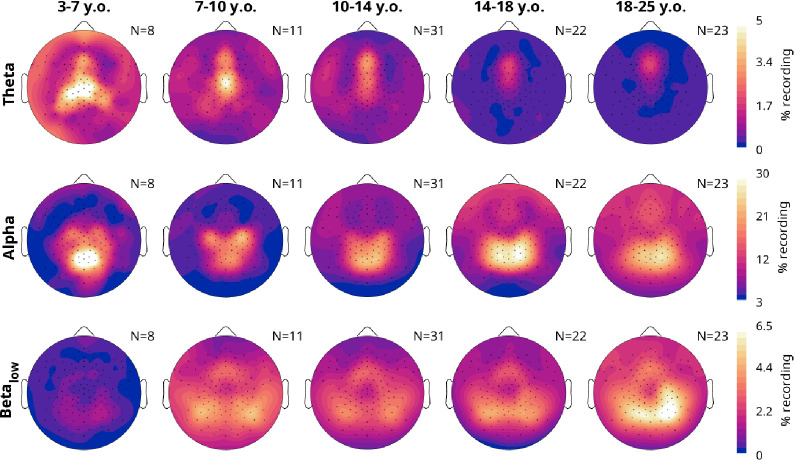
Average topographies of oscillation densities, split by frequency band. Recordings were from the oddball task, pooling controls and ADHD patients and evening and morning recordings. Theta is 4–7 Hz, alpha is 8–11 Hz, and low beta is 12–16 Hz.

Given the dissociation between alpha and beta for density, we explored the topographic changes in density, split by both age and frequency in Figure 6. Due to the drift in peak alpha frequency, and “bleeding” between bands, we chose to use non-adjacent frequency ranges to ensure independence: theta was from 4 to 7 Hz, alpha from 8 to 11 Hz, and low beta from 12 to 16 Hz.

Theta oscillations were the overall rarest. They were most prevalent in the youngest children at about 5% of the recording in central channels. With age, the peak in theta density gradually shifted upward and decreased in magnitude. Alpha density instead started as a midline occipital cluster in the 3–7-year-olds. Alpha densities in the occipital spot decreased in the 7–10 cohort, with bilateral central spots instead becoming more pronounced. With age, these three peaks morphed into a continuous occipital-parietal cluster, while in the remaining channels overall density of alpha increased. Lastly, low beta oscillations showed yet another topography. They were almost completely absent in the youngest group, and started to appear in the 7–10-year-olds as lateral occipital peaks and a frontal midline spot. Gradually, the lateral peaks converge towards the midline, and in adults became partially overlapping with alpha, on average right-lateralized.

**Figure 7:**
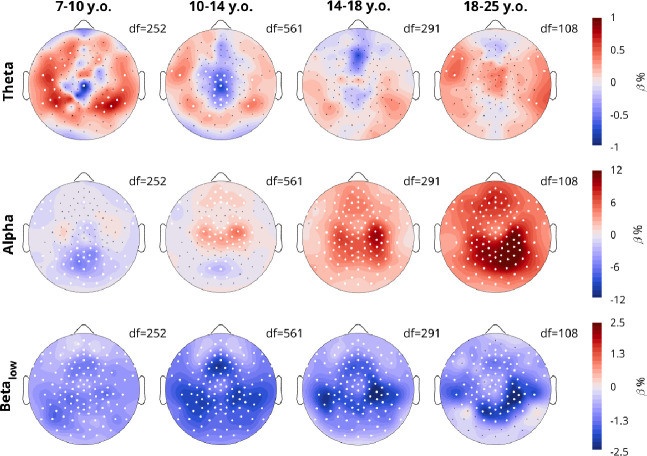
Topographies of overnight density changes, split by frequency band. Color indicates the β estimate for the linear mixed effects model, such that red indicates an overnight increase in density. The model was Density~Sleep+Task+(1|Participant)+(1|Participant:Session).

The overnight changes in density split by frequency are in Figure 7. Like in Figure 4, these reflect the β estimates for the fixed effect of Sleep, controlling for the fixed effect of Task, and mixed effects of Participant and Session.

The overnight changes in theta density were small (around 1%), however quite variable by region and age. In the 7–10-year-olds, theta density generally increased overnight except in a central spot, exactly where the largest theta densities were seen in Figure 6, which instead decreased. This continued for the 10–14-year-olds. In the 14–18-year-olds, there were no significant effects, however the frontal theta spot, now more frontal, showed a decrease. In adults, only some scattered theta increases were observed.

For alpha, the main occipital spot in the 7–10-year-olds decreased overnight. Already in the 10–14-year-olds a bilateral central alpha rhythm started to increase overnight, with still some slight decreases in the occipital spot. The overnight increases spread to the entire scalp in adolescents and adults, peaking in occipital parietal areas, especially right lateralized. For low beta, across all ages there are decreases, with the peak shifting across age bins.

### No EEG measure showed significant differences between ADHD and controls in any channel

**Figure 8:**
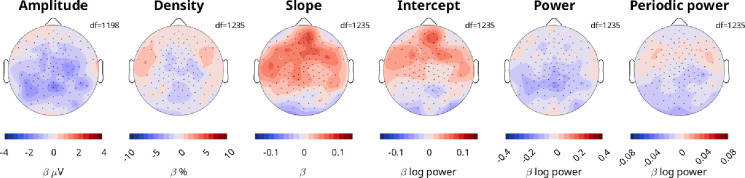
Effects of ADHD on EEG measures. Red indicates larger values in patients compared to controls. The scale for each topography is the same as for Figure 4. The model was Measure∼Sleep*Age+Task+Group+(1|Participant)+(1|Participant:Session). White dots would have indicated statistically significant channels, following FDR correction for multiple comparisons.

In our initial mixed effects models pooling channels and frequencies, we found no significant effects for ADHD, which is why in subsequent analyses and figures we no longer included a Group effect. However, we nevertheless conducted mixed effects models to determine the effect of ADHD for each channel, with fixed factors Group, Task, Sleep, Age, and Sleep by Age interaction, and nested mixed effects Participant and Session (Figure 8). We found no significant effects when correcting for multiple comparisons. However, amplitudes were on average lower in participants with ADHD and slopes were steeper compared to controls.

## DISCUSSION

In this study, we compared four measures of wake EEG data and their relationship to brain maturation, sleep, and ADHD: oscillation amplitudes and densities, and aperiodic slopes and intercepts. We hypothesized that oscillation amplitudes specifically would behave like sleep SWA. Our predictions were met on all accounts except for the sensitivity of amplitudes to ADHD. Of the four measures, only amplitudes decreased overnight in all ages *and* the overnight decrease was largest in younger children (Figure 2, Figure 4). Together with our previous results showing amplitudes increasing with time spent awake (Snipes, Meier, Meissner, et al., 2023), this indicates that wake amplitudes reflect the same information as sleep SWA: neuronal synchronization due to synaptic density and plasticity.

**Figure 9:**
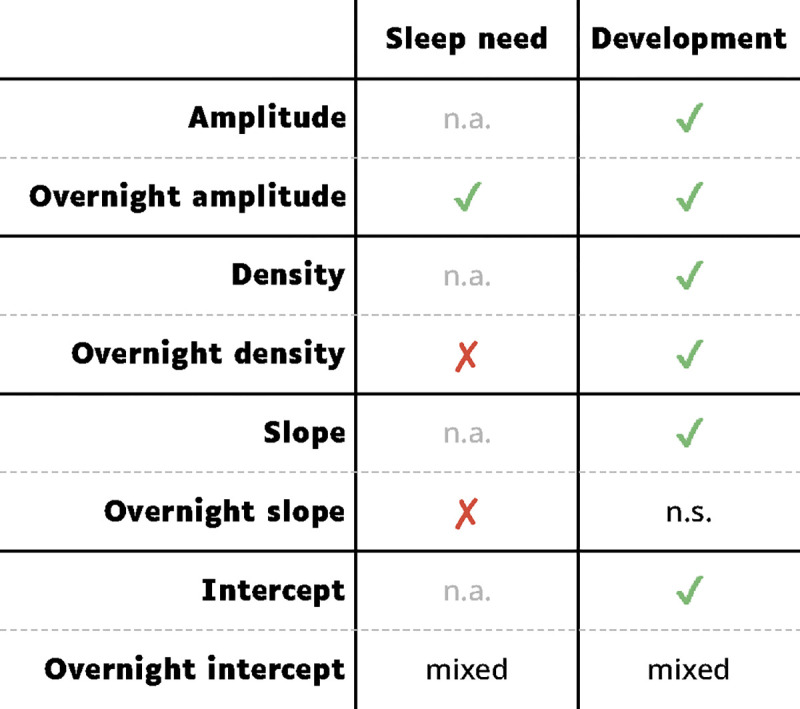
Summary of which outcome measure reflects which property. Sleep need was determined by whether an outcome measure decreased overnight and whether the decrease was larger in younger children. Development was determined by whether there were strong effects of age. Overnight intercepts had “mixed” results across analyses. Acronyms: n.a., not applicable; n.s., not significant.

While amplitudes were the only measure that followed the same patterns on all accounts as SWA, all EEG measures reflected brain development. Average amplitudes, slopes, and intercepts were all strongly anticorrelated with age, with intercepts showing the largest values (Figure 2). Average densities were not especially correlated with age, but we found distinct regional patterns with increasing age (Figure 6), indexing local differences across brain maturational stages. Overnight changes in both oscillation amplitudes and density were robustly correlated with age (Figure 2, Figure 7), whereas overnight changes in slopes and intercepts were not as affected by age, as evidenced by the low correlations in Figure 2. Finally, no measure showed significant effects of ADHD. These results are summarized in Figure 9.

The majority of EEG research relies on spectral power, with only a fraction dissociating periodic and aperiodic activity, and almost no research exists on wake oscillation bursts. Therefore, for comparability, we conducted the same analyses also on average power and periodic power. Furthermore, we correlated each outcome measure to power and periodic power (Suppl. Figure 1–1, Suppl. Table 1–1). Like this, we found repeatedly that average power from 4–16 Hz was most correlated with oscillation amplitudes, and average periodic power was mostly correlated with oscillation density, with comparable average and overnight topographies. This suggests that effects on power are more likely attributed to differences in oscillation amplitudes, while effects on periodic power are more likely due to differences in oscillation density. However, even with our own data this is not always the case; for example, the increase in central oscillation densities in adolescents corresponded to a decrease in periodic power (Figure 4), likely reflecting the relatively greater impact of the decrease in amplitudes. Furthermore, all outcome measures were significantly related to power even when controlling for participant and age (Suppl. Table 1–1). Whenever possible, these four EEG measures should be analyzed separately.

### Oscillation amplitudes

Matching our predictions, amplitudes decreased overnight, decreased with age, and the overnight decrease decreased with age. Like for SWA, the decrease in amplitudes with age can be explained by the decrease in synchronization due to reduced synaptic density in the cortex (Huttenlocher, 1979). Likewise, the overnight decrease in amplitudes could be because sleep reduces synchronization through net “synaptic down-selection” (Cirelli & Tononi, 2022; Tononi & Cirelli, 2014), and such plastic changes are more pronounced in children than adults (Jaramillo et al., 2020). Regarding regional effects, like for SWA in sleep (Kurth et al., 2010), we observed a posterior-anterior regional gradient in the overnight decrease in amplitudes, such that the decrease was more pronounced in occipital regions in children compared to adults (Figure 4). Across development, primary sensory and motor areas obtain peak cortical thickness earlier in children, followed by adjacent secondary areas and finally frontal association areas (Shaw et al., 2008), resulting in an overall posterior-anterior maturation trajectory. Therefore, younger children show larger overnight decreases in amplitudes in occipital areas because these areas undergo higher plastic changes at that maturational stage. Finally, even the significant sex difference was comparable to SWA (Mourtazaev et al., 1995), with wake oscillation amplitudes higher in females than males (although this was not an a-priori hypothesis of this study). This may reflect smaller heads and reduced skull thickness in females (Dijk et al., 1989).

Nevertheless, the effects we observe for wake amplitudes are not as large as those for sleep slow waves. SWA is substantially more pronounced in frontal areas in adults (Finelli et al., 2001), whereas overnight changes in wake amplitudes were more uniformly distributed across the scalp in adults (Figure 4). While we did observe on average lower amplitudes in patients with ADHD (Figure 8), this was not statistically significant. Furthermore, in adults the overnight decrease in amplitudes is near 0 μV (Figure 2), which is not the case for changes in SWA during sleep (Borbély, 1982). One possible explanation for this last point, based on our previous study (Snipes et al., 2023), is that the evening wake recordings fell within the wake maintenance zone. This is a circadian time window that begins 2–4 hours before bedtime, ends just before bedtime, and is characterized by increased alertness (Shekleton et al., 2013; Strogatz et al., 1987). We had found that oscillation amplitudes are significantly reduced in this window, counteracting the otherwise monotonic buildup in amplitudes that occurred throughout the day (Snipes et al., 2023). Therefore, the overnight change in amplitudes may be reduced by this window, and in adults the contrasting effect of the wake maintenance zone may be sufficient to equalize evening and morning amplitudes. In theory, all the outcome measures may have reduced (or enhanced) effects due to the wake maintenance zone, and therefore it will be important to collect more resting wake data at other timepoints throughout the day to dissociate these effects. Unfortunately, very little is known about the wake maintenance zone, and currently to our knowledge no study has investigated the wake maintenance zone in children, but there may be additional interactions between such circadian rhythms and age.

Overall, oscillation amplitudes reflect the same information as SWA. However, wake amplitudes appear less sensitive than sleep slow waves to factors such as ADHD, and possibly more affected by circadian or other factors. Therefore, amplitudes *can* be used to quantify sleep need, but sleep SWA is preferable when possible.

### Oscillation densities

As could be expected, the density of oscillations was the most complex outcome measure, with effects differing depending on age, sleep, topography, and oscillation frequency. While we initially aggregated measures across frequencies (Figure 3, Figure 4), it was readily apparent that important differences emerge when splitting densities by frequency band (Figure 5–Figure 7). Most notably, theta oscillation densities (4–7 Hz) increase or decrease overnight depending on the location, alpha oscillation densities (8–11 Hz) increase or decrease depending on age, and low beta densities (12–16 Hz) decrease overnight in all ages in all channels.

First, as seen in the absolute topographies of Figure 6, we found a subtle spatial dissociation between theta and alpha in young children (3–7 years old) to our knowledge not previously reported. This dissociation has been observed in spectral frequency, usually by contrasting conditions, but with minimal spatial resolution (Meyer et al., 2019; Orekhova et al., 2006), or without providing topographies for these age groups (Cellier et al., 2021). Theta power changes found during tasks in infants was localized more frontally than what we observe here (Meyer et al., 2019). We find the peak origin of theta to be just below Cz, and the peak source of alpha to be at Pz. However, given that alpha even off-peak is substantially more prevalent than theta, these theta oscillations can easily be missed.

Nevertheless, theta oscillations are most prevalent in early childhood, and decrease progressively with age, supporting previous results measuring relative theta power (Somsen et al., 1997). We further found that the peak in theta densities steadily drifts more frontally across childhood and adolescence. This frontal theta in adults is known to originate from the midline prefrontal cortex, and to be anti-correlated to the default mode network (Ishii et al., 2014; Michels et al., 2010; Scheeringa et al., 2008). Therefore, this drift in theta may reflect the steady maturation of both frontal cortices and the default mode network (Fan et al., 2021).

There is still unresolved contradictory evidence on the role of theta in adults (Snipes et al., 2022), without including the question of theta during development. On the one hand, theta is often associated with cognitive effort (Buzsáki, 2005; Cavanagh & Frank, 2014; Meyer et al., 2019; Mitchell et al., 2008), on the other, it is also associated with sleepiness (Finelli et al., 2000; Smith, 1938; Snipes et al., 2022) and fatigue (Arnau et al., 2021; Tran et al., 2020; Wascher et al., 2014). A possible resolution to this paradox is that there are distinct oscillations that originate from different circuits with different functions and just happen to occur at the same frequency. Our results in Figure 7 would support this, as the peak source of theta shows overnight *decreases*, whereas theta from the rest of the cortex instead shows overnight increases, even as the theta peak drifts more frontally. Alternatively, theta could reflect a general form of “idling rhythm” (Snipes et al., 2022; Snipes, Meier, Accascina, et al., 2023), originating from disengaged cortical areas, and what changes from evening to morning is which circuits tend to idle. This is supported by simultaneous EEG-fMRI studies that find theta activity anti-correlating with brain metabolism (Scheeringa et al., 2008). This would make theta functionally comparable to alpha (Laufs et al., 2003), differing only by source and frequency.

Like theta, alpha begins in young childhood as a midline spot (Figure 6). Two lateral central peaks become more defined at 7–10 years of age. These likely reflect sensorimotor mu rhythms which appear when motor activity is absent or even suppressed (Pfurtscheller et al., 2006; Pineda, 2005), and is already present in infants (Berchicci et al., 2011). We find that with age, they become topographically indistinguishable from occipital alpha, at least when recorded during an oddball task. These lateral central peaks are the first to show overnight increases in 10–14 year-olds, while the overnight decrease in the occipital midline spot becomes less prominent. The overnight increases then spread over bilateral parietal and occipital areas across adolescence and adulthood. This dissociation between overnight decreases in childhood and increases in adulthood, as well as the slight differences in topography, could suggest that occipital alpha is in fact qualitatively distinct in children and adults. However, these rhythms were previously considered functionally equivalent because also in infants alpha power increases with eyes closed compared to eyes open (Stroganova et al., 1999). More research is needed on the sources of these oscillations.

It is also possible that this dissociation in overnight changes is driven by some other difference with age, such as a longer window of sleep inertia in young children, longer sleep duration, or a shifted circadian rhythm compared to adults. Melatonin in the morning is elevated in children under 10, whereas older children and adolescents have morning melatonin levels comparable with the rest of the day (Attanasio et al., 1985). In adults, alpha power fluctuates with circadian rhythm and is therefore synchronized to melatonin levels (Cajochen et al., 2002). Therefore, it is possible that the dissociation of decreasing/increasing alpha originates from children and adults being at different phases of their alpha circadian rhythm in the morning. More research is needed into the circadian effects on the EEG during development.

### Aperiodic intercepts and slopes

Our results on intercepts and slopes replicate previous findings: they decrease linearly with age (Cellier et al., 2021; Hill et al., 2022; Tröndle et al., 2022) and originate from broad, primarily midline sources (Favaro et al., 2023). Most recently however, McSweeney et al. (2023) found a quadratic relationship between age and intercepts/slopes, such that they peaked at 5–7 years old (from a large population between 4 and 11 years old). Unfortunately, we do not have many participants in this age range, so it is possible the linear trends we observe do not continue for younger children. We did not replicate the finding of higher intercepts in females than males (Bódizs et al., 2021), but this could be due to the different age ranges of our study populations (3–25 vs. 17–60).

New to the literature is our finding of overnight changes. We find that slopes and intercepts go in opposite directions, with slopes becoming steeper and intercepts decreasing after a night of sleep. Curiously, when measuring aperiodic activity *during* sleep, slopes become progressively shallower across the night (Horváth et al., 2022). Otherwise, slopes and intercepts follow similar maturational changes in sleep as in wake, decreasing with age (Bódizs et al., 2021; Favaro et al., 2023). We find that the peak overnight change in slope is occipital (Figure 4), with the topography relatively stable across ages, and the effect decreasing slightly with age. Likewise, the decrease in intercepts after sleep was not especially strong and not especially dependent on age. Therefore, aperiodic activity during wake is not as sensitive to the interactions between sleep/wake history and development as oscillatory activity.

Intercepts, as expected, were highly correlated to slopes (Suppl. Figure 1–1). Any change in slope occurs at a pivot point, and when this point is not the intercept itself, it produces also a change in intercept. Likely the occipital overnight increases in intercepts observed in Figure 4 were driven rather by the increase in slopes, contrasting the general trend of a decrease in intercept. This interdependence between slopes and intercepts should be mitigated in future analyses. This can be done by identifying the pivot point where the slope changes. Bodizs et al. (2021) for example correlated slopes and intercepts calculated at every frequency, then took as “intercept” the power of the aperiodic signal where the correlation to slope was the lowest. Given that intercepts are strongly associated with age and with sleep/wake history, it would be advisable to dissociate them from slopes more systematically in order to tease apart their respective effects during development.

### ADHD

Despite a relatively substantial sample size (N=58), we did not observe any significant effects of ADHD on our EEG measures. We did find slopes to be steeper on average in patients, supporting the results of Robertson et al. (2019), and contrasting those of Ostlund et al. (2021). One explanation could be that our participants were performing tasks for most recordings, and the differences between patients and controls may mostly emerge in resting EEG; patients may have developed compensation mechanisms masking potential differences during the tasks. It’s also possible our analysis did not reach significance because our participants were a combination of both medicated and unmedicated patients, and it is known that medication will reduce the effects of ADHD on the EEG (Furrer et al., 2019; Karalunas et al., 2022). Additionally, our participants were screened for good sleep quality (to have a chance of falling asleep in the laboratory with an EEG net, and to have similar levels of sleep pressure as controls). However, around 40–55% of children with ADHD report sleep deficits (Becker et al., 2019; Corkum et al., 1998; Holmberg & Hjern, 2006; Konofal et al., 2010), so it is possible that poor sleep quality in patients contributes to differences in the wake EEG observed in prior studies (Clarke et al., 2020). Finally, it’s possible that only subtypes of patients with ADHD have a specific relationship to any of the measured EEG markers; ADHD is highly heterogeneous with varying symptoms among individuals.

Regardless of the reason, given that we do not see any systematic differences between patients and controls, none of the wake EEG outcome measures we tested make for a reliable intrinsic marker of ADHD which could potentially be used to aid diagnosis. Instead, research investigating such markers in these and other patient populations should take special care to control for sleep/wake history and sleep quality, as these may have a greater impact on the EEG. Instead, within the same study population we observed significant differences between controls and children with ADHD during sleep (Furrer et al., 2019), suggesting SWA during sleep is a more sensitive measure of developmental deficits.

### Limitations

The primary limitation of this study is the scarcity of datasets under 8. It is known that in young children there is a switch from primarily synaptic growth to primarily synaptic pruning, peaking in different cell populations and regions at different ages (Cao et al., 2020; Petanjek et al., 2011; Shaw et al., 2008), which is also reflected in SWA peaking in this period (Feinberg & Campbell, 2013). This would suggest more complex relationships between age and EEG outcome measures than the linear trends observed here.

Regarding interpretability, overnight changes do not dissociate between homeostatic (related to sleep need) and circadian (related to clock time) effects. To do so would require substantially more intensive protocols, involving sleep deprivation, restriction, or shifting sleep windows over several days. However, collecting more wake recordings throughout the day would already provide an indication as to when an effect is circadian or homeostatic.

Finally, our data is limited to EEG. Future studies and analyses would greatly benefit by comparing these outcome measures to structural and functional brain changes observable with MRI, as well as cognitive and behavioral outcome measures related to development. This would bridge the gap between a purely basic research finding to practical applications.

## Conclusions

We have found that overnight changes in oscillatory activity provide unique markers of brain maturation. Both absolute amplitudes and overnight changes in amplitudes decrease linearly with age, the effect more occipital in younger children. This makes wake amplitudes markers of brain plasticity and sleep need, just like sleep slow wave activity. Wake amplitudes are not as sensitive as sleep slow waves, but measuring sleep EEG is not always possible, therefore wake oscillation amplitudes are a useful alternative. Overnight changes in oscillation density, especially of alpha oscillations, dissociate children from adolescents and adults by switching from an overnight decrease to an increase in density. Understanding the reason behind this effect would likely provide important information on brain development around puberty and adolescence. More generally, we have shown that there are a multitude of changes in the EEG with development that go beyond simple spectral power, each with their own functional significance. Moving forward, researchers should analyze these measures individually, as they offer independent insights into neuronal activity.

## METHODS

### Datasets

The data for this manuscript was assembled from previous sleep studies conducted between 2008 and 2021, the primary focus of which had been the sleep EEG. The participant demographics of each dataset are in Table 1. In total, we included 163 participants between the ages 3.5 and 24.7, 38% female, 7% left-handed. Of these, 36% were diagnosed with ADHD at the department for Child and Adolescent Psychiatry at the University of Zurich, the outpatient clinic of the Child Development Center, and at private children’s clinics in Zurich Oerlikon. Patients were not excluded based on medication status, and therefore were a mixture of medicated, previously medicated, and unmedicated (see Table 2, and (Furrer et al., 2019; Ringli et al., 2013)). Otherwise, all participants were screened by telephone such that they all were completely healthy, took no (other) medication, had no (other) comorbidities, and were good sleepers. All participants were recruited from canton Zurich, Switzerland, and recorded at the University Children’s Hospital of Zurich, except for the children from 3.5 to 8 (Dataset2009), who were recruited in Providence, RI, USA, and recorded at home. All participants were recorded with high-density EEG. Sleep time was determined by their individual preferred sleep and wakeup time, which they had to maintain the week prior to each measurement. Wake measurements were done just before going to sleep, and ~30 minutes after waking up. 115 participants had 2 sessions, spaced at least 1 week apart, both included in these analyses. Depending on the dataset, different paradigms were used involving different wake tasks (described below). Therefore, there could be 1–4 recordings at each time point (morning/evening) in each session. In total, 1243 recordings were included in these analyses.

Informed consent was obtained from all adult participants, and from the legal guardians of all children below 14, as well as from adolescent participants 14–18. All studies were approved by the local ethics committees and performed according to the declaration of Helsinki.

### Oddball & motor adaptation paradigms

95 participants (Dataset2008, Dataset2009, Dataset2010, Dataset2016) performed an auditory oddball task during their wake EEG. The task lasted 4 minutes and was performed in the evening just before going to bed and in the morning ~30 minutes after waking up. The task involved 300 tones at ~80 dB, with an interstimulus interval of 0.8 s. A random 10% of stimuli were targets to which the participant had to push a button in response. For the Dataset2009 young children, the 4-minute task was split into 2 segments.

66 of these participants (Dataset2008, Dataset2010) also performed a half-hour visuomotor adaptation task (Ghilardi et al., 2000) followed by a second oddball. One dataset (Dataset2008) also included a second session with a control visuomotor task (no adaptation), counterbalanced with the motor adaptation task. The motor tasks were not included in this analysis, because they further differed from evening to morning. For more details on the adaptation task see Wilhelm et al. (2014). The youngest (Dataset2009) and oldest (Dataset2016) participants only conducted one oddball and no motor task, although the oldest also had two sessions.

The sleep data from these participants has been previously published (Buchmann et al., 2011; Furrer et al., 2019, 2020; Jaramillo et al., 2020; Kurth et al., 2010; Ringli et al., 2013; Volk et al., 2018; Wilhelm et al., 2014), as has a subset of the wake EEG data (Fattinger et al., 2017).

### Attention paradigm

68 participants (Dataset2017, Dataset2019) performed three tasks with a focus on attention. These were studies investigating the relationship between slow waves, behavior, and MR spectroscopy (Jaramillo et al., 2020; Volk et al., 2019). This included two sessions to compare the effects of phase targeted auditory stimulation on slow waves in sleep (sham and stimulation; data currently unpublished). The wake tasks were part of the Test Battery for Attentional Performance (TAP) (Zimmermann & Fimm, 2012), which included 2 minutes of a Go/No-Go task (respond to 1 stimulus, withhold response to another), 4.5 minutes of the Alertness task, and then 2 1.5-minute fixation recordings. For one dataset (Dataset2019), only 1 fixation recording was measured.

### EEG recordings and preprocessing

All datasets were measured using 128 channel EGI Geodesic Sensor nets and EGI amplifiers (Electrical Geodesics Inc., EGI, Eugene, OR, USA). Recordings were done with Cz reference, 1000 Hz sampling rate, and impedances kept below 50 kOhm. All analyses were performed in MATLAB 2023b, with the EEGLAB toolbox v2023.1 (Delorme & Makeig, 2004) and custom scripts.

EEG data was first mean-centered, then lowpass filtered at 40 Hz and notch-filtered at either 50 or 60 Hz (Dataset2009) along with subsequent harmonics. The data was downsampled to 250 Hz, then highpass-filtered over 0.5 Hz (Kaiser filter, stopband=0.25 Hz, stopband attenuation=60, passband ripple=0.05).

Artifacts were removed with a fully automated procedure. Movement and other large artifacts were detected in data filtered between 1 and 40 Hz, in 3 s segments. A segment was labeled a “major artifact” if it exceeded 500 μV, or a “minor artifact” if the correlation with neighboring channels was below .3. Major artifacts were always removed, either by removing all data in all channels during those 3 s, or removing the entire channel with such an artifact, depending on which (channel or segment) removed the least amount of clean data. Minor artifacts were removed in a similar way, removing iteratively either the channel with the most artifactual segments, or the segments with the most artifactual channels, until all channels and all segments had at most 30% of the data containing a minor artifact. Flat channels were removed using EEGLAB’s *clean_artifacts* function. Physiological artifacts (blinks, eye movements, muscle tone, heartbeat) were removed with independent component analysis (ICA), with components calculated separately as described in the next section. After these were removed, a second pass was conducted using EEGLAB’s *clean_windows()* function (MaxBadChannels=.3, PowerTolerances=[−inf, 12]), then bad segments/channels still containing amplitudes over 140 μV were removed, and finally EEGLAB’s *clean_channels_nolocs()* was applied (MinCorrelation=.5, IgnoredQuantile=.1, MaxBrokenTime=.5). Recordings for which more than 25 channels were removed, or which had less than 1 minute of data, were excluded from analysis. In a last step, EEG channels were interpolated, for a total of 123 channels, excluding the external electrodes (49 56, 107, 113) and the face electrodes (126, 127).

### Automatic detection and removal of physiological artefacts using ICA

For ICA, EEG data was first preprocessed as previously described, however the filtering was between 2.5 and 100 Hz, and the sampling rate was downsampled to 500 Hz. Automatically detected bad channels and bad time windows were removed, an empty Cz channel added, and then the data was re-referenced to the average of all channels. EEGLAB’s *runica* function was run with principal component analysis (PCA) rank reduction. Then, components were automatically classified with EEGLAB’s *iclabel*, as either brain, muscle, eye, heart, line, channel noise, or other. This function provides a probability score for each label from 0 to 1, so the label with the largest score for each component was taken. Components classified as muscle, eye or heart were removed. Of the remaining noise classifications (line, channel, other), due to poor classification accuracy, an additional step was implemented. Spectral power was calculated for each component, smoothed over 5 Hz. FOOOF (fitting oscillations one over f; (Donoghue et al., 2020)) was applied to the spectrum between 8 and 30 Hz. Unlike for the analysis (Figure 2), these values were not inverted; negative slopes indicate a decrease in power with increasing frequency. Components for which the spectral slope was shallower than −0.5 (so almost flat or even tilted positive), were considered noise and therefore excluded. Using the manually labeled components in an independent adult dataset (Snipes et al., 2022), we confirmed that this procedure was sufficiently comparable to human detection of artifactual components. We further confirmed that the outcome matched human component classification in a small subset of the children’s data as well. However, considering the trend towards −0.5 slopes observed in Figure 2, for future datasets with older participants we would recommend a higher threshold.

For the Dataset2009 cohort of <8-year-olds, given how little data there was and how many more movement artifacts, we chose to apply to same manual artifact rejection as in Snipes et al. (2022) to preserve as much data as possible.

### Burst detection

Oscillatory activity was quantified using cycle-by-cycle analysis to detect bursts of oscillations. Bursts were detected with the same procedure outlined in Snipes et al. (2023) and the same thresholds as in (Snipes, Meier, Accascina, et al., 2023). Briefly, EEG was narrow-band-pass filtered in overlapping ranges (2–6 Hz, 4–8 Hz…), from which zero-crossings were detected. Then, in the broadband filtered data (0.5–40 Hz), peaks were identified between the zero-crossings, and a cycle was considered an oscillation from positive to positive peak. A minimum number of consecutive cycles must meet a set of criteria (monotonicity, period consistency, amplitude consistency, shape consistency, etc.) for this to be considered a burst. Importantly, amplitude itself is never used as a threshold, as this would create a greater dependency between amplitude and density (such that a decrease in an amplitude threshold would result in an automatic increase in density).

Three sets of criteria were used. The first aimed to detect bursts relying on many low-threshold criteria (frequency in range of narrowband filter; PeriodConsistency=.5; AmplitudeConsistency=.4; FlankConsistency=.5; ShapeConsistency=.2; MonotonictyInTime=.4; MonotonicityInAmplitude=.4; ReversalRatio=.6; MinCycles=4). The second had fewer criteria with intermediate thresholds but a higher minimum number of cycles (PeriodConsistency=.6; AmplitudeConsistency=.6; MonotonicityInAmplitde=.6; FlankConsistency=.6; MinCycles=5). The third set had fewer criteria but stricter monotonicity thresholds (frequency in range of narrowband filter; PeriodConsistency=.7; FlankConsistency=.3; MontonocityInAmplitude=.9; MinCycles=3). These criteria were chosen a-priori based on manual tuning of the burst detection on an independent dataset of wake EEG in adults during sleep deprivation.

After bursts were detected in each channel separately, they were grouped into clusters to identify bursts that occurred simultaneously in multiple channels with roughly the same frequency. The frequency of bursts was calculated as the inverse of the average distance between negative peaks (1/period). Bursts for which the shorter one overlapped at least 50%, and were within 1 Hz of each other, were considered part of the same burst cluster. Bursts identified separately in each channel were used for all the topographies, otherwise burst clusters were used to reduce the effect of burst globality (spread across the scalp) on measures of density.

### Oscillatory outcome measures

*Oscillation amplitudes* were calculated as the average negative to positive peak voltage difference for all cycles involved in all bursts, with units in microvolts (μV). *Oscillation densities* were calculated as the percentage of the recording occupied by bursts (sum of all the bursts’ durations divided by the duration of the recording). When calculating across multiple channels (e.g. Figure 2), oscillation density could easily exceed 100%, as burst clusters in different frequency ranges often co-occur. When combining densities across multiple frequency bands, bursts were pooled rather than averaged. In our previous publication (Snipes, Meier, Meissner, et al., 2023), we referred to oscillation densities as “quantities”, however this term did not properly account for the normalization in time.

### The choice of frequency bands

Only bursts between 2 and 16 Hz were detected. Below 4 Hz very few bursts could be identified, therefore only bursts above 4 Hz were included in the analysis. Bursts over 16 Hz could be detected, but with higher false-positive rates, as determined by visual inspection. The choice of cutoff at 16 Hz was done arbitrarily a-priori to capture alpha (8–12 Hz) with generous padding. The division of bands for Figure 6 and Figure 7 was done using conventional bands with 1 Hz gaps to reduce overlapping information due to the drift in peak frequencies across individuals and ages. The inclusion of low beta (12–16 Hz) was done based on results observed in Figure 5. The frequency range 12–16 Hz is traditionally called “sigma” in sleep research, but this is often used interchangeably with sleep spindles, therefore we opted for “low beta” to avoid ambiguity.

Many researchers advocate for the use of an individual alpha frequency (IAF) to define frequency ranges (Bazanova & Vernon, 2014; Klimesch, 1999; Tröndle et al., 2022). The shift in IAF with age makes a strong case for such an approach. However, the first problem with using IAF is the assumption that all frequencies will be adjusted together, so slower alpha means slower theta; essentially assuming individuals have intrinsically slower or faster brains. The second problem is that it assumes the peak oscillation will be functionally the same for all participants. We did not find compelling evidence yet that these assumptions were safe to have, and therefore we preferred to use fixed bands with gaps.

### Spectral power analysis

Spectral power was calculated using MATLAB’s *pwelch* function, with 4 s Hanning windows and 50% overlap. To dissociate periodic and aperiodic spectral power, we used the MATLAB extension of FOOOF. Spectra were smoothed over 2 Hz, and the aperiodic signal was fitted between 2 and 35 Hz, otherwise the default settings were used.

### Outcome measures

*Power* was calculated by averaging the log-transformed power values between 4 and 16 Hz. *Intercepts* were provided by FOOOF as the power value at 1 Hz of the aperiodic signal, and *slopes* as the exponents that describe the angle of the aperiodic signal. The values are inverted, such that positive slopes refer to a downward descending aperiodic signal, and the larger the value the steeper the descent. *Periodic power* was calculated as the log-transformed power, subtracting the aperiodic signal.

### Statistics

Statistics were performed using the MATLAB Statistics and Machine Learning Toolbox. For all analyses, statistical significance was determined for p-values < .05. Given the heterogeneous datasets pooled together for this analysis, we chose to conduct linear mixed effects models to model the relationship between age, sleep, ADHD and EEG measures. This was done with the function *fitlme()*.

In a first instance, we ran the following model on the EEG measures derived from the average of all channels and frequencies: Measure∼Task+Sleep*Age+Group+Sex+(1|Participant)+(1|Participant:Session). *Task* included the levels *Oddball* vs. *go/no-go*, *alertness*, and *fixation*. *Sleep* determined the main effect of *evening* vs. *morning*, and the interaction of age was one of the main hypotheses being tested. *Group* compared typically developing participants vs. those with ADHD. *Sex* compared females vs. males. Given that there were only minor effects, significant only for amplitude, this factor was not included in later models. We then included the random effect of session nested in the random effect of participant. While more elaborate models with a better fit could have been used, this model reflected our a-priori hypotheses that we aimed to test. As a simpler quantification of these effects, and sanity check, we conducted Pearson’s correlations between age and each measure, including only auditory oddball recordings, averaging sessions (Figure 2).

To determine the relationship of EEG measures to each other (Suppl. Figure 1–1, Suppl. Table 1–1), we conducted Pearson’s correlations across all recordings. Then, to control for the effects of sleep, age, session and task, we conducted linear mixed effects models with the following formula: Measure1∼Measure2+Sleep*Age+Task+(1|Participant)+(1|Participant:Session).

To determine the topographical distribution of the overnight effects (Figure 4, Figure 7), we ran simple linear mixed effects models on participants binned by age: Measure∼Sleep+Task+(1|Participant)+(1|Participant:Session). For the age bin of 18–25 year olds, without multiple tasks, the fixed effect of Task was excluded. We then plotted the β estimates for the effect of Sleep and their associated statistical significance, which was corrected for multiple comparisons across channels using false discovery rates (FDR; (Benjamini & Yekutieli, 2001)).

To determine the effect of ADHD across channels, we used the model Measure∼Task+Sleep*Age+Group+(1|Participant)+(1|Participant:Session) for each channel and plotted in Figure 8 the β estimates for the effect of Group.

## Supplementary Material

Supplement 1

## Figures and Tables

**Table 1:** Demographics, split by dataset. The year for each dataset indicates the beginning of data collection. N indicates the number of participants. Paradigm indicates which set of wake tasks were recorded. Sessions indicates the number of sessions expected for each dataset, although in practice due to drop-outs, some participants only completed 1.

	N #	Female %	Lefties %	ADHD %	Age range (years)	Mean age (years)	Paradigm	Sessions #

Dataset2008	38	32	0	0	8.7–23.4	14.1 (3.8)	Adaptation	2
Dataset2009	11	73	0	0	3.5–8.0	5.6 (1.5)	Oddball	1
Dataset2010	28	21	14	100	9.7–16.3	12.7 (1.9)	Adaptation	1
Dataset2016	18	44	0	0	18.4–24.7	21.6 (2.1)	Oddball	2
Dataset2017	42	43	17	36	8.1–17.6	12.2 (2.7)	Attention	2
Dataset2019	26	38	0	58	8.8–16.8	11.4 (2.0)	Attention	2
All	163	38	7	36	3.5–24.7	13.2 (4.4)		

**Table 2:** ADHD demographics, split by patient status.

	N #	Female %	Lefties %	Age range (years)	Mean age (years)	Oddball %

Medicated in the past (1)	6	17	0	9.7–14.8	12.5 (2.2)	33
Unmedicated (2)	14	21	14	9.5–15.3	11.3 (1.6)	64
Medication the day before (3)	22	23	14	8.7–16.3	12.3 (2.2)	45
Medication the day of (4)	13	15	0	9.0–16.1	12.3 (2.0)	54
**All patients**	**55** *	**20**	**9**	**8.7–16.3**	**12.1 (2.0)**	**51**
**Controls**	**105**	**47**	**6**	**3.5–24.7**	**13.8 (5.2)**	**58**

* For 3 patients, medication status was missing, the true total is therefore 58. The percentage of participants performing the oddball task includes both Adaptation and Oddball paradigms from Table 1.

## Data Availability

The burst detection can be conducted either with the original python toolbox bycycle (https://github.com/bycycle-tools/bycycle), or with our MATLAB implementation (https://github.com/HuberSleepLab/Matcycle). The preprocessing and analysis code is likewise open source (https://github.com/snipeso/children-wake/). The data is available upon reasonable request.
