# Supplementary material for: Wake EEG oscillation dynamics reflect both sleep need and brain maturation across childhood and adolescence
Source: bioRxiv. 2024 Feb 28:2024.02.24.581878. Preprint. [Version 1] doi: 10.1101/2024.02.24.581878 (PMC10925212; doi:10.1101/2024.02.24.581878)
Supplement: Supplementary file 1 [file NIHPP2024.02.24.581878v1-supplement-1.pdf]

bioRxiv preprint doi: https://doi.org/10.1101/2024.02.24.581878; this version posted February 28, 2024. The copyright holder for this preprint (which was not certified by peer review) is the author/funder, who has granted bioRxiv a license to display the preprint in perpetuity. It is made available under aCC-BY 4.0 International license.

SUPPLEMENTARY MATERIAL

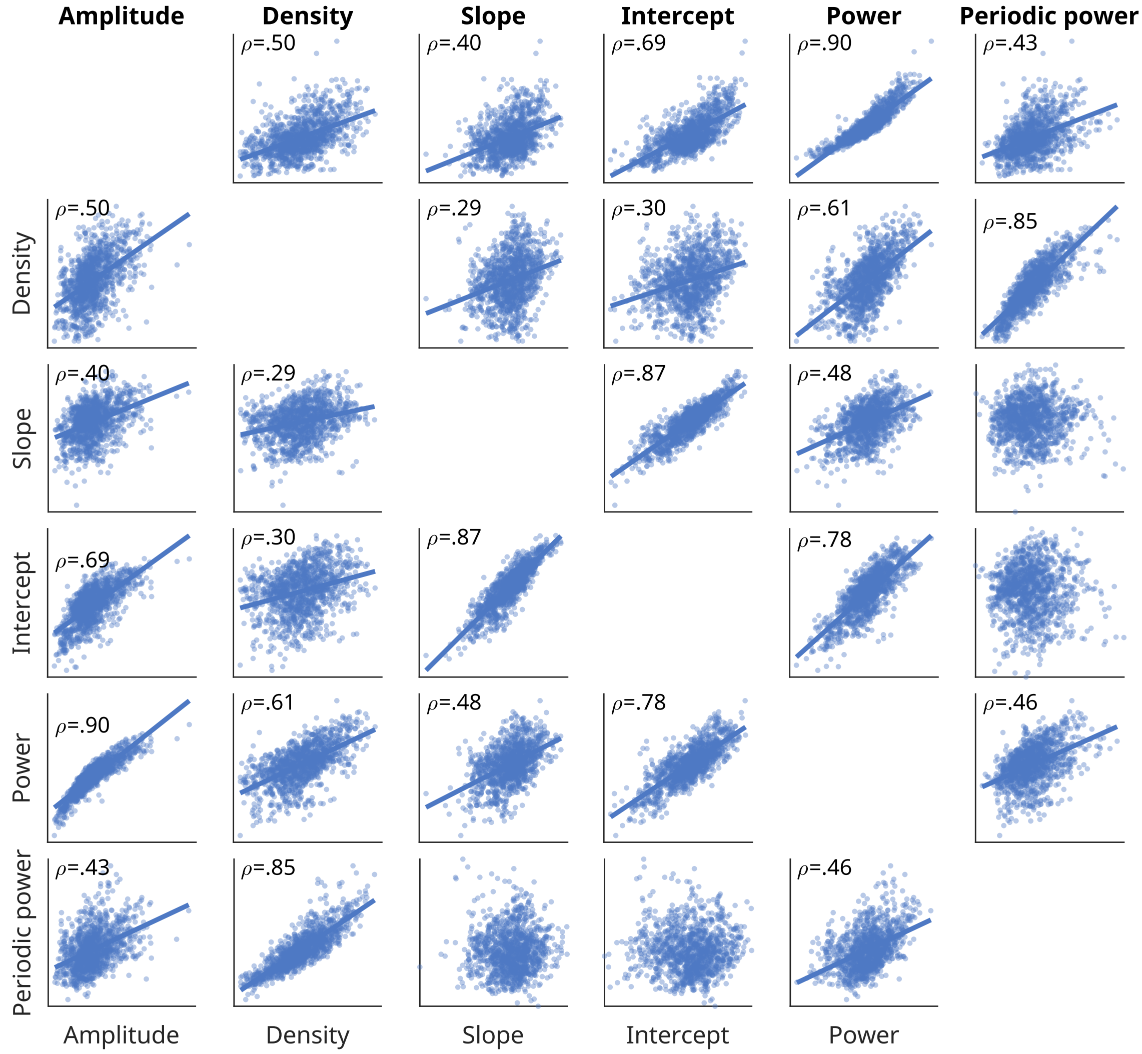

Supplementary Figure 1-1: Correlations between outcome measures, corrected for age. Each plot correlates one measure to the other (plots are mirrored along the diagonal). Each dot represents the data of a recording, with multiple recordings (morning/evening, session1/session2, etc) for each participant. Pearson's rho values are provided for correlations which were significant (p-value < .05). N.B. these correlations violate the assumption of independence between data points, and are merely an indication of how each measure relates to the other.

bioRxiv preprint doi: https://doi.org/10.1101/2024.02.24.581878; this version posted February 28, 2024. The copyright holder for this preprint (which was not certified by peer review) is the author/funder, who has granted bioRxiv a license to display the preprint in perpetuity. It is made available under aCC-BY 4.0 International license.

	AMPLITUDE	DENSITY	SLOPE	INTERCEPT	POWER	PERIODIC POWER
**AMPLITUDE**		b=10.00<br>t=13.2<br>p<.001<br>df=1235	b=-0.00<br>t=-0.5<br>p=.618<br>df=1235	b=0.02<br>t=8.7<br>p<.001<br>df=1235	b=0.07<br>t=39.3<br>p<.001<br>df=1235	b=0.02<br>t=20.6<br>p<.001<br>df=1235
**DENSITY**	b=0.01<br>t=12.0<br>p<.001<br>df=1235		b=0.00<br>t=14.7<br>p<.001<br>df=1235	b=0.00<br>t=14.5<br>p<.001<br>df=1235	b=0.00<br>t=28.2<br>p<.001<br>df=1235	b=0.00<br>t=46.9<br>p<.001<br>df=1235
**SLOPE**	b=-0.76<br>t=-1.9<br>p=.063<br>df=1235	b=167.38<br>t=14.9<br>p<.001<br>df=1235		b=0.88<br>t=61.9<br>p<.001<br>df=1235	b=0.18<br>t=4.7<br>p<.001<br>df=1235	b=0.17<br>t=12.5<br>p<.001<br>df=1235
**INTERCEPT**	b=3.05<br>t=7.6<br>p<.001<br>df=1235	b=160.06<br>t=14.5<br>p<.001<br>df=1235	b=0.84<br>t=61.0<br>p<.001<br>df=1235		b=0.63<br>t=17.5<br>p<.001<br>df=1235	b=0.12<br>t=8.4<br>p<.001<br>df=1235
**POWER**	b=7.54<br>t=40.5<br>p<.001<br>df=1235	b=181.32<br>t=29.9<br>p<.001<br>df=1235	b=0.10<br>t=5.8<br>p<.001<br>df=1235	b=0.32<br>t=19.4<br>p<.001<br>df=1235		b=0.22<br>t=30.2<br>p<.001<br>df=1235
**PERIODIC POWER**	b=13.77<br>t=19.3<br>p<.001<br>df=1235	b=666.33<br>t=46.7<br>p<.001<br>df=1235	b=0.61<br>t=12.1<br>p<.001<br>df=1235	b=0.46<br>t=8.5<br>p<.001<br>df=1235	b=1.73<br>t=27.9<br>p<.001<br>df=1235	

Supplementary table 1-1: mixed effects models between EEG measures. The model was $Measure_{column}$ ~ $Measure_{row}$ + Task + Sleep*Age + (1|Participant) + (1|Participant:Session). β estimates indicate how much each unit of the row measure changes each unit of the column measure (for a hypothetical evening oddball recording at age 0). Degrees of freedom (df) reflect the overall number of datapoints included in the model, minus the number of factors included in the model.
